# Sirtuin functions and modulation: from chemistry to the clinic

**DOI:** 10.1186/s13148-016-0224-3

**Published:** 2016-05-25

**Authors:** Vincenzo Carafa, Dante Rotili, Mariantonietta Forgione, Francesca Cuomo, Enrica Serretiello, Gebremedhin Solomon Hailu, Elina Jarho, Maija Lahtela-Kakkonen, Antonello Mai, Lucia Altucci

**Affiliations:** Dipartimento di Biochimica, Biofisica e Patologia Generale, Seconda Università degli Studi di Napoli, Vico L. De Crecchio 7, 80138 Napoli, Italy; Dipartimento di Chimica e Tecnologie del Farmaco, “Sapienza” Università di Roma, 00185 Roma, Italy; Center for Life Nano Science@Sapienza, Istituto Italiano di Tecnologia, Viale Regina Elena, 291, 00161 Roma, Italy; School of Pharmacy, University of Eastern Finland, FI-70210 Kuopio, Finland; Istituto Pasteur, Fondazione Cenci-Bolognetti, “Sapienza” Università di Roma, 00185 Roma, Italy; Istituto di Genetica e Biofisica, Via P. Castellino 111, 80131 Napoli, Italy

**Keywords:** Epigenetics, Sirtuins, SIRT modulators, Drug discovery, Cancer, Neurodegeneration

## Abstract

Sirtuins are NAD^+^-dependent histone deacetylases regulating important metabolic pathways in prokaryotes and eukaryotes and are involved in many biological processes such as cell survival, senescence, proliferation, apoptosis, DNA repair, cell metabolism, and caloric restriction. The seven members of this family of enzymes are considered potential targets for the treatment of human pathologies including neurodegenerative diseases, cardiovascular diseases, and cancer. Furthermore, recent interest focusing on sirtuin modulators as epigenetic players in the regulation of fundamental biological pathways has prompted increased efforts to discover new small molecules able to modify sirtuin activity. Here, we review the role, mechanism of action, and biological function of the seven sirtuins, as well as their inhibitors and activators.

## Background

The mammalian sirtuin (SIRT) family, evolutionally conserved proteins belonging to class III histone deacetylases (HDACs), comprises seven members. SIRTs share a NAD^+^-binding catalytic domain and may act specifically on different substrates depending on the biological processes in which they are involved (Table 1). SIRTs differ in sequence and length in both their N- and C-terminal domains, partially explaining their different localization and functions. SIRTs can catalyze both deacetylation and ADP-ribosylation [1]. Their best-characterized activity is NAD^+^-dependent lysine deacetylation, but recent studies demonstrated that some SIRTs also remove other acyl groups such as succinyl, malonyl, glutaryl, and long-chain fatty acyl groups [2, 3] (Table 1). The last decade has seen growing interest in their association with and involvement in different pathologies, such as (but not restricted to) cancer and neurodegenerative diseases. Increasing evidences supporting the potential use of SIRT inhibitors (SIRTi) for the treatment of cancer, HIV infection, and muscular diseases and of SIRT activators (SIRTa) for age-related disorders have led to the identification of many SIRT modulators over recent years, mainly through chemical library screening and catalytic mechanism-based design approaches, often combined with structure-activity relationship (SAR) investigations. Here, we provide a summary of the most important SIRT activities and SIRT modulators, their latest analogs, and novel chemotypes (Tables 2 and 3).

**Table 1:** Sirtuin location, activity, and effects on pathologies

Sirtuin	Localization	Enzymatic activity	Histone deacetylation target	Non-histone deacetylation target	Pathology
SIRT1	Nuclear/cytoplasmatic	Deacetylase	H3K9ac	Hif-1α, Hif-2α	Neurodegenerative diseases. Cancer: acute myeloid leukemia, colon, prostate, ovarian, glioma, breast, melanoma, lung adenocarcinoma.
			H1K26ac	MYC	
			H4K16ac		
SIRT2	Nuclear/cytoplasmatic	Deacetylase	H3K56ac	Tubulin	Neurodegenerative diseases. Cancer: brain tissue, glioma.
			H4K16ac	Foxo3a	
				EIF5A	
				P53, G6PD, MYC	
SIRT3	Mitochondrial	Deacetylase	H3K56ac	SOD2, PDMC1a, IDH2, GOT2, FoxO3a	Neurodegenerative diseases. Cancer: B cell chronic lymphocytic leukemia, mantle cell lymphoma, chronic lymphocytic leukemia, breast, gastric.
			H4K14 ac		
SIRT4	Mitochondrial	ADP-ribosyltransferase	Unknown	GDH, PDH	Cancer: breast, colorectal.
SIRT5	Mitochondrial	Malonyl, succinyl, glutaryl deacetylase	Unknown	CPS1	Cancer: pancreatic, breast, non-small cell lung carcinoma.
SIRT6	Nuclear	Deacetylase, ADP-ribosyltransferase, long-chain fatty acyl deacylase	H3K9ac	Unknown	Cancer: breast, colon.
			H3K56ac		
SIRT7	Nuclear	Deacetylase	H3K18ac	Hif-1α, Hif-2α	Cancer: liver, testis, spleen, thyroid, breast.

**Table 2:** Most relevant sirtuin inhibitors

Compound	Structure	Enzyme activity	Biological Effects	Reference(s)
**1** *,* splitomicin		ySir2 IC_50_ = 60 μM SIRT1: no inhibition @500 μM	Minimal inhibitory concentration (MIC) in yeast of 0.49 μM.	[37]
**2**, HR-73		SIRT1 IC_50_ = < 5 μM	Decreased HIV transcription through viral Tat protein acetylation.	[38]
**3, 4**		**3** SIRT2 IC_50_ = 1.5 μM **4** SIRT2 IC_50_ = 1.5 μM (racemate) SIRT2 IC_50_ = 1.0 μM (*R* enantiomer)	Weak antiproliferative effects and increased α-tubulin acetylation in MCF-7 breast cancer cells.	[39]
**5**, EX-527, selisistat		SIRT1 IC_50_ = 0.098 μM SIRT2 IC_50_ = 19.6 μM SIRT3 IC_50_ = 48.7 μM	Induction of p53 acetylation in different cell lines. Modulation of the acetylation status of the mutant huntingtin via SIRT1 inhbition shown to restore transcriptional dysregulation in models of Huntington’s disease (HD) treatment. In a first Phase II clinical trial found to be safe and well tolerated in early stage HD patients at plasma levels within the therapeutic concentration range in preclinical HD models.	[40–44]
**6**, cambinol		SIRT1 IC_50_ = 56 μM SIRT2 IC_50_ = 59 μM SIRT3: no inhibition @300 μM SIRT5: 42% inhibition @300 μM	Induces apoptosis in BCL6-expressing Burkitt lymphoma cells. Reduces tumor growth of Burkitt lymphoma in a mouse xenograft model. Reduces neuroblastoma formation in *N*-Myc transgenic mice.	[45–50]
**7**, AGK2		SIRT1 IC_50_ > 50 μM SIRT2 IC_50_ = 3.5 μM SIRT3 IC_50_ > 50 μM	Protective against Parkinson’s disease (PD) in different PD models. Induces caspase-3-dependent apoptosis and necrosis of C6 glioma cells.	[24, 51]
**8**, tenovin-1 **9**, tenovin-6		**Tenovin-1**: IC_50_s not determined due to lack of water solubility **Tenovin-6**: SIRT1 IC_50_ = 21 μM SIRT2 IC_50_ = 10 μM SIRT3 IC_50_ = 67 μM	**Tenovin-6** Cytotoxic to the ARN8 melanoma cells. Delayes the growth of xenograft tumors derived from ARN8 cells. Decelerates the disease progression of chronic myelogenous leukemia in mice model. Potent antitumor activity in various human gastric cancer cells via death receptor 5 (DR5) upregulation.	[52–54]
**10**, sirtinol		ySir2 IC_50_ = 70 μM SIRT1 IC_50_ = 131 μM SIRT2 IC_50_ = 49 μM	Inhibits the viability of MCF-7, H1299, DU145, PC3 and HeLa cells. Enhances the chemosensitivity to camptothecin and cisplatin in DU145, PC3 and HeLa cells. Promotes a signifcant growth inhibition or apoptosis in cells from adult T-cell leukemia-lymphoma (ATL) and HTLV-1-related cell lines.	[55–59]
**11**, salermide		SIRT1 IC_50_ = 43 μM SIRT2 IC_50_ = 25 μM	Induces apoptosis in various cancer lines (mainly MOLT4, KG1A, SW480, and Raji) but not in normal cells (MRC5) through inhibition of SIRT1 in a p53-independent manner. Induces apoptosis in NSCLC cells through upregulation of CDR5. Exerts potent antiproliferative effects on MOLT4, MDA-MB-231 and RKO cancer cells and in colorectal carcinoma (CRC) and glioblastoma multiforme (GBM) cancer stem cells (CSCs). Exerts cell protection effects in a *C. elegans* model of muscular dystrophy. Induces apoptosis and death of schistosomula, separation of adult worm pairs and reduction in egg laying, through inhibition of the *S. mansoni* specific sirtuin	[60–64]
**12**, MC2141		SIRT1 IC_50_ = 9.8 μM SIRT2 IC_50_ = 12.3 μM	Induces apoptosis in U937 cells. Exerts significant antiproliferative effects on Raji , DLD1, HeLa, and CRC and GBM CSCs.	[65, 66]
**13**, inauhzin		SIRT1 IC_50_ = 0.7- 2 μM SIRT2: no inhibition @50 μM SIRT3: no inhibition @50 μM	Activates and stabilizes p53 by inhibiting SIRT1 activity with consequent p53 acetylation and prevention of MDM2-mediated p53 ubiquitylation. Inhibits cell proliferation and promotes senescence and tumor-specific p53-dependent apoptosis without genotoxicity in different cell lines (mainly H460, HCT116, and A549 cells). Represses the growth of xenograft tumors derived from p53-harbouring H460 and HCT116 cells.	[67]
**14**		SIRT1 IC_50_ = 4 nM SIRT2 IC_50_ = 1 nM SIRT3 IC_50_ = 7 nM	Not reported	[68]
**15**, SirReal2		SIRT2 IC_50_ = 0.14-0.44 μM SIRT1 22% inhibition @100 μM SIRT3 no inhibition @100 μM SIRT4 no inhibition @200 μM SIRT5 no inhibition @200 μM SIRT6 19% inhibition @200 μM	Hyperacetylation of α-tubulin and of the microtubule network in HeLa cells (@20 μM). Significant depletion of the SIRT2 substrate BubR1 (spindle assembly checkpoint protein) in HeLa cells. No alteration of cell cycle distribution in HeLa cells.	[69]
**16-20**		**16** SIRT2 IC_50_ = 0.18 μM SIRT1 29% inhibition @200 μM **17** SIRT2 IC_50_ = 0.26 μM SIRT1 no inhibition @200 μM **18** SIRT2 IC_50_ = 0.21 μM SIRT1 no inhibition @200 μM **19** SIRT2 IC_50_ = 0.31 μM SIRT1 no inhibition @200 μM **20** SIRT2 IC_50_ = 0.32 μM SIRT1 no inhibition @200 μM	Hyperacetylation of α-tubulin and of the microtubule network in HeLa cells (**16** @10 μM).	[70]
**21**		SIRT2 IC_50_ = 48.3 nM SIRT1 IC_50_ = 12 μM SIRT3 IC_50_ = 44.2 μM	Time-dependent and dose-dependent hyperacetylation of α-tubulin in MCF-7 cells. Modest cytotoxic effects (CC_50_ 26-33μM) in human cancer cell lines (MCF-7, K562 and DU145).	[71]
**22, 23**		**22** SIRT2 IC_50_ = 3.7 μM **23** SIRT2 IC_50_ = 12.2 μM SIRT1/3 no inhibition @200 μM for both compounds	Dose-dependent hyperacetylation of α-tubulin in MCF-7 cells. Dose-dependent antiproliferative effects on MCF-7 and A549 cancer cells.	[72, 73]
**24**, AK-7		SIRT2 IC_50_ = 15.5 μM SIRT1/3 no inhibition @50 μM	Neuroprotective reduction of total neuronal cholesterol biosynthesis in a SIRT2 inhibition-dependent way. Neuroprotective effects in different models of Huntington’s disease and Parkinson’s disease.	[74–76]
**25**, SDX-437		SIRT3 IC_50_ = 700 nM SIRT1 no inhibition @100 μM	Not reported.	[77]
**26**		SIRT6 IC_50_ = 89 μM SIRT1 IC_50_ = 1578 μM SIRT2 IC_50_ = 751 μM	Hyperacetylation of SIRT6 substrate H3K9 in pancreatic adenocarcinoma BxPC-3 cells. Glucose uptake increase, dose-dependent upregulation of GLUT-1 expression and reduction of TNF-α secretion in BxPC-3 cells.	[78]
**27, 28**		**27** SIRT1 IC_50_ = 3.9 μM **28** SIRT1 IC_50_ = 2.7 μM SIRT2 IC_50_ = 23 μM SIRT3 IC_50_ > 100 μM	Not reported.	[84, 85]
**29**		SIRT1 IC_50_ = 0.9 μM SIRT2 IC_50_ = 3 μM SIRT3 IC_50_ = 8 μM	Antiproliferative effect in A549 lung carcinoma and MCF-7 breast carcinoma cell lines.	[86, 87]
**30**		SIRT1 IC_50_ = 6 μM SIRT2 IC_50_ = 26 μM SIRT3 IC_50_ = 29 μM	Antiproliferative effect in A549 lung carcinoma and MCF-7 breast carcinoma cell lines.	[86, 87]
**31-33**		**31** SIRT1 IC_50_ = 11 μM SIRT2 IC_50_ = 77 μM **32** SIRT1 IC_50_ = 12 μM SIRT2 IC_50_ = 30 μM **33** SIRT1 IC_50_ = 0.7 μM SIRT2 IC_50_ = 4 μM	Not reported.	[88]

**Table 3:** Most relevant sirtuin activators

Compound	Structure	Enzyme activity	Biological Effects	Reference(s)
**34**, resveratrol		SIRT1 EC_1.5_^*a*^ = 46.2 μM	Improves mitochondrial functions and protects against fat diet-induced obesity (DIO). In obese mice leads to increased health-span and lifespan. Promising effects and in clinical trials for the treatment of some diseases of aging (metabolic disorders, type 2 diabetes, etc.).	[91–96, 106–109]
**35-37**		**35** SIRT1 EC_1.5_ = 2.9 μM SIRT1 max act.^*b*^ 447% **36** SIRT1 EC_1.5_ = 0.16 μM SIRT1 max act. 781% **37** SIRT1 EC_1.5_ = 0.36 μM SIRT1 max act. 296%	All SRT compounds, with different potencies, improve insulin sensitivity in DIO and genetically obese mice (Lep^*ob/ob*^), lower plasma glucose and increase mitochondrial capacity. In Zucker *fa/fa* rats, improve whole-body glucose homeostasis, insulin sensitivity in adipose tissue, skeletal muscle and liver, and mitochondrial bioenergetics in a SIRT-1 dependent manner. Very good activities in different age-related disease conditions.	[95, 97–101]
**38**, STAC-8		SIRT1 EC_1.5_ = 1.2 μM SIRT1 max act. 890%	Promising activities in different age-related disease models: obesity and metabolic disorders, inflammatory and autoimmune disorders, cardiovascular disease, hepatic steatosis, neurodegeneration, and cancer.	[99–101]
**39**		SIRT1 EC_1.5_ = 0.5 μM SIRT1 max act. 220%	Not reported.	[99–102]
**40, 41**		**40** SIRT1 EC_1.5_ = 0.3 μM SIRT1 max act. 253% **41** SIRT1 EC_1.5_ = 0.4 μM SIRT1 max act. 820%	Not reported.	[99–102]
**42-44**		EC_1.5_ < 250 nM SIRT1 max act. ≥300%	Not reported.	[99–101, 103]
**45**, STAC-5		SIRT1 EC_1.5_ = 0.4 μM SIRT1 max act. 1310%	Promising activities in different age-related disease models: obesity and metabolic disorders, inflammatory and autoimmune disorders, cardiovascular disease, hepatic steatosis, neurodegeneration, and cancer.	[99–101, 104]
**46**		**46a** (MC2562) (Ar = Ph, R_1_ = OEt, R_2_ = benzyl): SIRT1 EC_1.5_ ≈ 1 μM SIRT2 EC_1.5_ = 25 μM SIRT3 EC_1.5_ ≈ 50 μM	Induce α-tubulin hypoacetylation in U937 cells and reduce the number of senescent cells of 30-40% in human mesenchymal stem cells. Increase NO release in HaCat cells, and accelerate tissue renewal in a mouse model of skin repair. In C2C12 myoblasts improve mitochondrial density and functions through activation of the SIRT1/AMPK axis. A water-soluble analog displays antiproliferative effect and increased H4K16 deacetylation in a panel of human cancer cells at 8-35 μM	[111–113]
**47**, honokiol		Increases SIRT3 levels by nearly two-fold @5μM and 10μM in cardiomyocytes after 24h treatment. *In vitro* directly binds to SIRT3 and increases the affinity of SIRT3 for NAD^+^.	Blocks hypertrofic response of cardiomyocytes *in vitro*. Protects mice from developing cardiac hypertrophy, attenuates pre-established cardiac hypertrophy in mice, reduces ROS production and prevents cardiomyocyte death in a SIRT3-dependent manner.	[114]

^*a*^Concentration of compound required to increase enzyme activity by 50 %

^*b*^Maximum percentage of activation achieved at the highest tested dose

## Sirtuins in physiology and pathology

SIRT1 was the first SIRT family member to be discovered and is still the most studied. Its involvement in many neuronal processes [4, 5] prompted further investigations into its role in neurological disorders such as Alzheimer’s disease (AD), Parkinson’s disease (PD), and Huntington’s disease (HD) [6–8]. SIRT1 exerts a neuroprotective action and is involved in survival, neuropathology, and the expression of brain-derived neurotrophic factor. In a mouse model of HD, SIRT1 knockout results in exacerbation of the pathology, while its re-expression displays neuroprotective effects [9]. A correlation between nicotinamide phosphoribosyltransferase (NAMPT) and SIRT1 was recently shown. NAMPT is a therapeutic target against ischemic stroke, acting in vascular repair and neurogenesis. SIRT1-mediated deacetylation of NAMPT at K53 increased its activity and secretion [10]. The first observations linking SIRT1 to tumorigenesis came from two studies showing p53 deacetylation and inhibition [11, 12], promoting cancer cell death. In tumorigenesis, SIRT1 seems to play a contradictory role, acting as both a tumor promoter and tumor suppressor (by inhibiting oncogenes and oncoproteins, similar to survivin) [13, 14]. SIRT1 regulates many tumor suppressors and DNA repair genes [15–18]. SIRT1 upregulation is described in many human malignancies [19]. Although its role in cancer remains contradictory [20], recent studies showed that patients expressing high levels of SIRT1 had a higher chance of being resistant to chemotherapy than patients with low SIRT1 expression [21, 22]. One report proposed a role for SIRT1 in melanoma and suggested the use of inhibitors (tenovins, EX-527, and sirtinol), either alone or in combination. These inhibitors act on a variety of SIRTs, strengthening the idea that the concomitant inhibition of several SIRTs may contribute to a reduction in malignant growth [23]. SIRT2, the second member of the SIRT family, seems to exert a neurodegenerative action in neurological disease [24]. Indeed, pharmacological or genetic inhibition of SIRT2 blocks of αSyn-mediated toxicity [25]. SIRT2 is involved in apoptosis control through p53 process regulation. Several studies confirm the role of SIRT2 in the control of cell cycle progression at many levels. It was demonstrated that it is a checkpoint for metaphase/anaphase processes and G2/M transition [26, 27]. Recent studies indicated that SIRT2 might also act as a tumor promoter or tumor suppressor in tumorigenesis, likely displaying a regulatory function [28, 29]. Moreover studies about SIRT2 have shown its involvement in metabolism processes, like adipogenesis [30]. SIRT2 is capable to deacetylate the Glucokinase (GCK), an essential enzyme for maintaining homeostasis of glucose regulated by the binding of the glucokinase regulatory protein (GKRP). Acetylated GKRP is connected to diabetes mellitus [31]. Of the three mitochondrial SIRTs (SIRT3, 4, and 5), some evidence correlates SIRT3 with neurodegenerative disease. SIRT3 protects neurons in the cochlea against oxidative damage during calorie restriction [32] and in response to stress-regulating mitochondrial antioxidant manganese superoxide dismutase (MnSOD) in microglia [33]. A number of studies also hypothesize its involvement in cancer progression. SIRT3 mainly inhibits mitochondrial ROS production but also deacetylates and activates many mitochondrial proteins, regulating proliferation, differentiation, and survival [34, 35]. Recent data suggest that SIRT3 acts as a tumor suppressor by inhibiting glycolysis metabolism after the deacetylation and activation of pyruvate dehydrogenase; thus, also the role of SIRT3 in cancer is debatable. Much less is known about the remaining SIRTs. Unlike other family members, SIRT4 lacks NAD^+^-dependent deacetylase activity but exerts ADP-ribosyltransferase activity on histones. SIRT4 is induced by DNA damage including chemotherapy and γ-irradiation and is capable of arresting the cell cycle by inhibiting mitochondrial glutamine metabolism. SIRT4 inhibits proliferation, invasion, and migration in colorectal cancer cells, and its low expression is correlated with a worse prognosis [36]. SIRT5 is reported to interact with carbamoyl phosphate synthetase 1 (CPS1), and it is deacetylated by SIRT5. The role of SIRT5 in carcinogenesis remains unclear, but a recent study showed its overexpression in non-small cell lung cancer tissues as a marker of low survival [37]. SIRT6 was reported to display actions controlling cellular homeostasis, DNA repair, telomere maintenance, and metabolism, acting as an epigenetic guardian for cellular differentiation [38]. Recent studies have highlighted SIRT7 as a promising target for epigenetic cancer therapy. SIRT7 catalyzes selective deacetylation of H3K18, an emerging epigenetic biomarker of aggressive tumors, controlling many tumor suppressor genes. High expression of SIRT7 has in fact been associated with aggressive cancers and low survival, whereas its depletion leads to a less aggressive phenotype [39]. A recent report described the use of chlorpromazine to inhibit cell cycle progression in rat glioma cells and induce autophagic cell death. The authors demonstrate that an FDA-approved drug used to treat schizophrenia and bipolar disorder is able to increase p53 Lys382 acetylation through a mechanism of SIRT1 inhibition [40].

## SIRT inhibition—small molecules

Many SIRT modulators, used alone or in combination with other epigenetic modulators or known drugs, have been described as having beneficial effects against neurodegeneration and cancer [41]. Identified in 2001 from a yeast-based screening for inhibitors of Sir2, splitomicin (**1**, Table 2), despite being substantially inactive against hSIRTs, has been the starting point for the development of several SIRT1/2 inhibitors [42]. Among them, the compound with a phenyl ring on 2-position and a bromine on 8-position indicated as HR-73 (**2**, Table 2) is a single-digit micromolar SIRT1i that inhibits HIV transcription through affecting the Tat protein acetylation [43]. In a series of splitomicin analogs carrying a phenyl ring on 3-position and different substituents on 8-position, compounds **3** and **4** (Table 2), respectively, revealed to be low micromolar SIRT2i able to exert antiproliferative effects and cause α-tubulin hyperacetylation in MCF-7 cells [44]. Discovered in 2005, EX-527 (selisistat, **5**, Table 2) was the first potent, selective (over SIRT2/3), and cell-permeable SIRT1i [45, 46]. Despite increasing p53 acetylation, EX-527 has no major effect on viability and proliferation in various tumors [47]. EX-527 is being developed for HD. A phase II clinical study recently demonstrated that EX-527 is safe and well tolerated in HD patients at plasma concentrations, providing benefit in non-clinical HD models [48]. Recently, EX-527 was also found to block the amplification of human papillomavirus via a SIRT1 inhibition-dependent mechanism [49]. Identified in 2006, cambinol (**6**, Table 2) is a moderate SIRT1/2i that induces hyperacetylation of p53, α-tubulin, FOXO3a, and Ku70 in NCI-H460 and HeLa cancer cells; promotes apoptosis in BCL-6-expressing Burkitt lymphoma cells; and reduces tumor growth in a xenograft model [50]. It is also endowed with anticancer effects on hepatocellular carcinoma tumor models in vitro and in vivo [51] and reduces neuroblastoma formation in *N*-Myc transgenic mice [52]. These promising results stimulated various optimization efforts, leading to the identification of derivatives/analogs with increased potency/selectivity for either SIRT1 or SIRT2 [53–55]. Discovered through a focused library screening, AGK2 (**7**, Table 2) is a single-digit micromolar SIRT2i selective over SIRT1/3. Able to inhibit the deacetylation of α-tubulin in HeLa cells, AGK2 rescues dopaminergic neurons from α-synuclein toxicity and protects against PD in different PD models [25]. Likely through SIRT2 inhibition, AGK2 also induces caspase-3-dependent death in glioma cells [56]. A cell-based screen designed to detect p53 activators identified tenovin-1 (**8**, Table 2) and its water-soluble analog tenovin-6 (**9**, Table 2) as SIRTi. Tenovin-6 is a micromolar SIRT1-3i that increases the level of p53K382ac; shows cytotoxic effects on melanoma cells, delaying the growth of ARN8-derived xenograft tumors [57]; and induces apoptosis in gastric cancer cells [58] and in chronic myeloid leukemia cells, decelerating the disease progression in mice models [59]. Discovered in 2001 by a high-throughput cell-based screening, sirtinol (**10**, Table 2) is a micromolar ySir2 and hSIRT1/2 inhibitor endowed with various anticancer activities [60]. It induces senescence-like growth arrest in human MCF-7 and H1299 cells [61]; inhibits the growth of PC3, DU145, and HeLa cells, enhancing their chemosensitivity to cisplatin and camptothecin [62, 63]; and promotes significant growth inhibition or apoptosis in cells from adult T cell leukemia-lymphoma (ATL) [64]. Salermide (**11**, Table 2), which resulted from the med-chem optimization of sirtinol, is a moderate SIRT1/2i that induces cancer-specific pro-apoptotic effects on different tumor cells (mainly MOLT4, KG1A, SW480, and Raji) through reactivation of pro-apoptotic genes (*CASP8*, *TNF*, *TNFRSF10B*, and *PUMA*) previously repressed by SIRT1-mediated H4K16ac deacetylation [65] and through the upregulation of *DR5* [66]. Salermide and its analogs also show potent antiproliferative effects on cancer stem cells [67] and exert cell protection effects on a *C. elegans* model of muscular dystrophy [68]. In infectious diseases (*Schistosoma mansoni*), salermide was the most potent among the tested SIRTi in inducing apoptosis and death of schistosomula, in separation of adult worm pairs and in reduction in egg laying, through inhibition of the *S. mansoni* specific sirtuin [69]. Identified in 2010 as result of cambinol manipulation, MC2141 (**12**, Table 2) is the prototype of a series of benzodeazaoxaflavins active as low micromolar SIRT1/2i. MC2141 displays significant pro-apoptotic and antiproliferative properties in different cancer cell lines, including cancer stem cells [70, 71]. Identified in 2012 in a virtual screening for inhibitors of the p53-MDM2/MDMX interaction, inauhzin (**13**, Table 2) is a (sub)micromolar SIRT1i selective over SIRT2/3 able to exert antiproliferative and tumor-specific pro-apoptotic effects on various cancer lines by activating and stabilizing p53. Inauhzin represses the growth of xenograft tumors derived from p53-harboring H460 and HCT116 cells [72]. The most potent SIRTi reported to date are thieno[3,2-*d*]pyrimidine-6-carboxamides that resulted from a SAR analysis of the hit compounds deriving from a DNA-encoded small molecule library screen [73]. Although the most potent compound in the series (**14**, Table 2) is active in the single-digit nanomolar range against SIRT1–3 and its binding mode has been elucidated by X-ray crystallography, no biological activities are currently known for these compounds. Very recently, SirReal2 (**15**, Table 2) was reported as a submicromolar SIRT2i with >1000-fold selectivity over SIRT1/3/4/5/6 [74]. Its potency and selectivity, as revealed by X-ray crystallography, are based on a ligand-induced structural rearrangement of the SIRT2 active site revealing a previously unexploited binding pocket. The SIRT2 inhibition capability of SirReal2 has been confirmed in HeLa cells by the induction of α-tubulin hyperacetylation and the destabilization of the SIRT2 substrate BubR1. A subsequent SAR investigation on the prototype led to the development of some derivatives (**16**–**20**, Table 2) with improved SIRT2i potency [75].

An elegant fragment-based approach inspired by the structures of the SIRTi suramin and nicotinamide recently identified a nanomolar SIRT2-selective (over SIRT1/3) inhibitor (**21**, Table 2), which is able to induce clear time-dependent and dose-dependent hyperacetylation of α-tubulin in MCF-7 cells and shows cytotoxic effects on some cancer cell lines [76]. In recent years, a series of chroman-4-one derivatives as low micromolar SIRT2i selective over SIRT1/3 has been reported [77, 78]. Among them, compounds **22** and **23** (Table 2) are the most attractive in terms of their overall pharmacological profile as they display good dose-dependent α-tubulin hyperacetylation and significant antiproliferative effects on cancer cells (MCF-7 and A549). From the screening of a panel of polyglutamine aggregate inhibitors, AK-7 (**24**, Table 2) was identified in 2011 as a brain-permeable micromolar SIRT2 selective (over SIRT1/3) inhibitor able to reduce neuronal cholesterol biosynthesis [79]. In addition, AK-7 displays other SIRT2 inhibition-dependent neuroprotective effects on various models of HD [80] and PD [81], supporting the development of SIRT2i as potential therapeutics for these disorders. Very recently, by high-throughput screening employing self-assembled monolayer desorption/ionization mass spectrometry, the first submicromolar small molecule inhibitor of SIRT3, SDX-437 (**25**, Table 2), was identified. SDX-437 is >100-fold selective over SIRT1 and could be a useful tool for studying SIRT3 biology and a promising lead for future med-chem optimization campaigns [82]. By a high-throughput molecular docking screen, compound **26** (Table 2) was recently identified as the first micromolar small molecule inhibitor of SIRT6 with >eightfold selectivity over SIRT1/2 [83]. Although this molecule needs optimization, it can be considered as a promising lead for the functional annotation of SIRT6 and the development of potential SIRT6-based therapeutics due to its ability to induce hyperacetylation of the SIRT6 substrate H3K9, to increase glucose uptake through the upregulation of GLUT-1, and to reduce TNF-α secretion (BxPC-3 cells).

## SIRT inhibition—peptides and pseudopeptides

The first peptidic SIRTi was developed by replacing the *N*^ε^-acetyl-lysine with a thioacetylated residue and by using the C-terminus of p53 protein (H_2_N-KKGQSTSRH(ThAcK)LMFKTEG-COOH) as a substrate peptide template [84]. Based on kinetic studies, this thioacetylated peptide was able to form an intermediate tighter than the acetylated peptide [84–86], showing a strong SIRT1 inhibition (IC_50_ ~ 2 μM), and was also active against SIRT2/3 (IC_50_ ~ 2 and 67 μM, respectively) [87]. More thioacetylated peptide inhibitors were developed from other SIRT substrates such as human α-tubulin and acetyl-coenzyme A synthetase 2 (AceCS2) [87, 88]. The α-tubulin [85–93]-based H_2_N-MPSD(ThAcK)TIGG-COOH inhibited SIRT2 at a low micromolar level (IC_50_ ~ 11 μM), whereas it was less potent against SIRT1/3. The thioacetylated AceCS2 (633–652) peptide showed low micromolar inhibition of SIRT1–3 (IC_50_ ~ 0.9, 4, and 5 μM, respectively) [87]. Thioacetylated α-tubulin and p53-based tri-, tetra-, and pentapeptides were developed in 2009 [88]. Several peptides had submicromolar IC_50_s for SIRT1, such as tripeptide K(ThAcK)L (0.57 μM) and pentapeptide HK(ThAcK)LM (0.18 μM). The most potent inhibitor for SIRT2 was the pentapeptide HK(ThAcK)AM (IC_50_ ~ 3.8 μM). The first small pseudopeptidic SIRTi was reported in 2009 [89]. The most potent compound, NCS-12k (**27**, Table 2), showed an IC_50_ of 3.9 μM for SIRT1. Further modifications produced the cell-active Cbz-Lys(ThAc)-NH-Ph (**28**, Table 2) with an IC_50_ of 2.7 μM for SIRT1, 23 μM for SIRT2, but interestingly >100 μM for SIRT3 [90]. Among other *N*^ε^-thioacetylated pseudopeptide inhibitors developed to improve potency, selectivity, and cell permeability, compounds **29** and **30** (Table 2) were found to be micromolar inhibitors of SIRT1-3 and showed antiproliferative effects on cancer cells [91, 92]. Some pseudopeptidic inhibitors were also tested against SIRT6, but potency was lower [93]. Besides the thioacetyl group, several other lysine *N*^ε^-modifications (such as propionyl, butyryl, α-hydroxyacetyl, monofluoroacetyl, homocitrulline, homoarginine, difluoroacetyl, acetimidoyl, methanesulfonyl, selenoacetyl, and trifluoroacetyl groups) have been reported [85, 86, 93–95]. Among the carbonyl-containing *N*^ε^-modifications studied on the sequence Ac-AKA-OH, the most potent were *N*^ε^-3,3-dimethylacryl (**31**, Table 2) and *N*^ε^-isovaleryl (**32**, Table 2) with IC_50_s in the micromolar range against SIRT1/2, while *N*^ε^-isothiovaleryl (**33**, Table 2) improved potency giving SIRT1/2 IC_50_s in the (sub)micromolar range [95].

## SIRT activators

The natural polyphenol resveratrol (**34**, Table 3) was the first SIRT1a described. Its administration extends lifespan in yeast, *Caenorhabditis elegans*, *Drosophila*, fishes, and bees [96–99]. Mice treated with resveratrol display improved mitochondrial functions and protection against high-fat diet-induced obesity [100], while in obese mice, treatment with resveratrol leads to increased healthspan and lifespan [101]. Identified in 2007 by high-throughput screening as selective SIRT1a more potent than resveratrol, SRT compounds (**35**–**37**, Table 3) reproduce most of resveratrol beneficial effects in vivo. In diet-induced and genetically obese (Lep^*ob*/*ob*^) mice, compounds **35**–**37** (Table 3) improve insulin sensitivity, lower plasma glucose, and increase mitochondrial capacity. Moreover, in Zucker (*fa*/*fa*) rats, the same compounds improve whole-body glucose homeostasis and insulin sensitivity [95]. Due to their very promising activities, some of the most potent SRTs are currently in clinical trials for the treatment of different age-related diseases [102–106]. The most potent scaffolds proposed as sirtuin-activating in terms of SIRT1 activation (EC_1.5_ values in the submicromolar range) and the most effective in the treatment of age-related conditions (obesity, metabolic and cardiovascular disorders, inflammatory/autoimmune diseases, neurodegeneration, and cancer) are pyrido[3,2-*b*][1, 4]oxazocines (**38**, Table 3) [104–106]; oxazolo[4,5-*b*]pyridines and benzo[*d*]imidazoles (**39**–**41**, Table 3) [104–107]; thiazolopyridines (**42**–**44**, Table 3) [104–106, 108]; and azabenzimidazoles (**45**, Table 3) [104–106, 109]. Over the past years, it has been highly debated the effective SIRT1 activation by resveratrol and SRT compounds, because only the use in the enzyme assays of aminomethylcoumarin (AMC)- or carboxytetramethylrhodamine (TAMRA)-labeled substrates allowed to see activation, whereas in the presence of natural substrates no activation was observed [110–112]. For this reason, it was questioned if the action of resveratrol and SRT compounds on SIRT1 is the result of a direct activation, or if rather it arises from the indirect modulation of other pathways, such as AMPK activation and/or PDE inhibition [113]. Recently, direct SIRT1 activation has been confirmed, and it has been shown that there are subtle structural and positional requirements to detect SIRT1 activation with some of its natural substrates (e.g., FOXO3a and PGC-1α) [104, 114]. Moreover, E230K or E230A SIRT1 mutation abolishes enzyme activation as well as binding of SRT compounds, suggesting an assisted allosteric activation mechanism in which the activators bind and stabilize the enzyme-substrate complex promoting the deacetylation reaction [104]. Lastly, the crystal structure of a SRT compound bound to an engineered minimally functional hSIRT1 has been reported, unambiguously confirming the direct allosteric activation of SIRT1 by small molecules [115]. The 1,4-dihydropyridine (DHP) scaffold with general formula **46** (Table 3) has been described as a new chemotype for SIRT activation. Selected DHPs induced hypoacetylation of α-tubulin in U937 cells, high NO release in HaCat cells, and ameliorated skin repair in a mouse model of wound healing. In myoblasts, they improved mitochondrial density and functions through activation of the SIRT1/AMPK axis. A water-soluble analog displayed antiproliferative action and increased H4K16ac deacetylation in a panel of cancer cells at 8–35 μM [116–118]. Very recently, the cardiac anti-hypertrophic effects of the natural compound honokiol (**47**, Table 3) were reported to depend on the pharmacological activation (increased expression and activity) of SIRT3 [119].

## Conclusions

A critical analysis of current knowledge and recent discoveries clearly indicates that SIRT modulation is beneficial against several diseases including cancer and neurodegeneration. Chronic diseases, such as obesity and diabetes, might also likely benefit from SIRT targeting, but further investigation is needed. The ubiquitous expression of some SIRTs, together with the fact that many studies primarily investigated SIRT1 only, and not all the other family members, still leaves room for some confusion. Currently, for example, activation or inhibition of SIRTs (even the same SIRT) is described as beneficial for pathologies such as neurodegeneration. Despite the apparent contradiction, this is theoretically possible when taking into account the role of SIRT-containing complexes and not just the catalytic domain of the single enzyme. Furthermore, both the chemistry and biomedical characterization of SIRTa are at a much earlier stage of development than SIRTi, thus suggesting that new alternatives may arise in the near future. Finally, the potential selective modulation of SIRT family members will represent a promising area provided that new selective molecules can be designed.
